# Are Copy Number Variations within the *Fec^B^* Gene Significantly Associated with Morphometric Traits in Goats?

**DOI:** 10.3390/ani12121547

**Published:** 2022-06-15

**Authors:** Yi Bi, Zhiying Wang, Qian Wang, Hongfei Liu, Zhengang Guo, Chuanying Pan, Hong Chen, Haijing Zhu, Lian Wu, Xianyong Lan

**Affiliations:** 1 College of Animal Science and Technology, Key Laboratory of Animal Genetics, Breeding and Reproduction of Shaanxi Province, Northwest A&F University, Xianyang 712100, China; biyi0312@163.com (Y.B.); wangzhiying9779@163.com (Z.W.); wangqian21028@163.com (Q.W.); lhf563@nwafu.edu.cn (H.L.); chuanyingpan@126.com (C.P.); chenhong1212@263.net (H.C.); lxw784@alumni.bham.ac.uk (L.W.); 2 State Key Laboratory of Livestock Biology, Northwest A&F University, Xianyang 712100, China; 3 Bijie Animal Husbandry and Veterinary Science Research Institute, Bijie 551700, China; 4 Shaanxi Provincial Engineering and Technology Research Center of Cashmere Goats, Yulin University, Yulin 719000, China; haijingzhu@yulinu.edu.cn

**Keywords:** copy number variation, goat, *Fec^B^* gene, morphometric traits

## Abstract

**Simple Summary:**

The *Booroola* fecundity (*Fec^B^*) gene, as a major fertility-related gene in sheep, is currently attracting considerable attention. While first discovered in sheep, determining whether FecB is a vital gene in goats has recently become a major research interest. Contemporary research has addressed this issue and discovered that several *Fec^B^* variations in goats are significantly associated with litter size in goats. Given that growth development is positively associated with litter size, *Fec^B^* may also exert a significant effect on goat morphometric traits. Five copy number variations (CNVs) of the *Fec^B^* gene were tested in Shaanbei white cashmere (SBWC, *n* = 318), Guizhou Heima (GZHM, *n* = 203), and Nubian (*n* = 120) goats, all of which were significantly associated with body morphometric traits in the Shaanbei white cashmere goats (*p* < 0.05). Notably, individuals with the normal type of CNV3 consistently displayed a superior phenotype in litter size and morphometric traits, which allows its use as an effective marker for goat breeding.

**Abstract:**

The *Booroola* fecundity (*Fec^B^*) gene is a major fertility-related gene first identified in Booroola sheep. Numerous studies have investigated whether the *Fec^B^* gene is a major fecundity gene in goats or whether there are other genes that play a critical role in goat fertility. Nevertheless, little attention has been paid to the role of the *Fec^B^* gene in the body morphometric traits of goats, despite the positive relationship discerned between litter size and growth. We identified five copy number variations (CNVs) within the *Fec^B^* gene in 641 goats, including 318 Shaanbei white cashmere (SBWC) goats, 203 Guizhou Heima (GZHM) goats, and 120 Nubian goats, which exhibited different distributions among these populations. Our results revealed that these five CNVs were significantly associated with goat morphometric traits (*p* < 0.05). The normal type of CNV3 was the dominant type and displayed superior phenotypes in both litter size and morphometric traits, making it an effective marker for goat breeding. Consequently, LD blocks in the region of 10 Mb upstream and downstream from *Fec^B^* and potential transcription factors (TFs) that could bind with the CNVs were analyzed via bioinformatics. Although no significant LD block was detected, our results illustrated that these CNVs could bind to growth-related TFs and indirectly affect the growth development of the goats. We identified potential markers to promote litter size and growth, and we offer a theoretical foundation for further breeding work.

## 1. Introduction

The *Booroola* fecundity gene (*Fec^B^*), also known as the bone morphogenetic protein receptor-1B *(BMPR1B)* gene, is the first major gene discovered that is related to sheep fertility [1,2,3,4,5]. *Fec^B^* is also involved in granulosa cell differentiation, follicle development, and ovulation in sheep [6,7]. The missense mutation A746G in the *Fec^B^* gene, which causes these effects, might lead to a loss of gene function, thereby increasing progesterone release, promoting follicle maturation, and increasing ovulation rate [8,9,10]. Moreover, ewes with the homozygous mutant GG genotype g.746A > G (p.Q249R) have significantly increased litter sizes compared to those with other genotypes [11]. The discovery of *Fec^B^* has raised the question of whether it exerts other physiological effects on regulating fecundity in sheep. In Booroola sheep, luteinizing hormone (LH) levels are significantly higher in individuals with the g.746A > G mutant than in wild-type individuals at different periods and physiological states. These mutant-type individuals also have higher levels of follicle-stimulating hormone (FSH) released by the pituitary cells in contrast to wild-type individuals [12]. Additionally, ewes with g.746A > G have nonseasonal polyestrus. Over 60% of ewes have repeated estrus in winter and summer, whereas the frequency of repeated estrus in g.746A > G ewes during the non-breeding season is only about 20–30% [13].

Although *Fec^B^* is partially responsible for sheep fecundity, its role in goats remains unknown. Recently, this issue has received increasing attention, and several studies have revealed fertility-related mutations in goats [14,15]. For example, five copy number variations (CNVs) within the *Fec^B^* gene were detected in Shaanbei white cashmere (SBWC) goats, two of which significantly influenced litter size [16]. Moreover, two novel single nucleotide polymorphisms (SNPs) (A775G and G777A) were confirmed in Markhoz goats; however, this requires further investigation [17]. In Liaoning cashmere goats, C94T within *Fec^B^* reportedly had a significant effect on litter size, and the litter size increased in the presence of CC and CT genotypes [18]. Additionally, T242C was identified in seven Indian goat breeds but without any significant association with litter size [19]. Since growth development is positively associated with litter size [20], determining whether *Fec^B^* is also related to morphometric traits in goats is vital; however, no current research has focused on this issue.

Therefore, we aim to detect five CNVs within the *Fec^B^* gene, which were identified in our previous study on Shaanbei white cashmere (SBWC), Guizhou Heima (GZHM), and Nubian goats, and to assess their distributions and associations with morphometric traits via molecular experiments and bioinformatic analyses. Notably, these findings clarify whether *Fec^B^* can improve goat morphometric traits and pave the way for further breeding work.

## 2. Materials and Methods

All experiments in this study were approved by the Northwest A&F University (IACUCNWAFU; protocol number NWAFAC1008). In addition, the permission of the ethics committee was obtained to use the experimental animals in the study.

### 2.1. Sample Collection

Ear tissue samples were collected randomly from a total of 641 goats, including 318 SBWC, 203 GZHM, and 120 Nubian goats. Each population was raised under the same conditions and was deemed healthy. Moreover, almost all the goats had records of morphometric traits, including body weight, height and length, chest depth and width, heart girth, and cannon bone circumference [21].

Genomic DNA was isolated from ear tissues using phenol-chloroform [21], and the extracted genomic DNA was qualified using a Nanodrop 1000 (Thermo Scientific, Waltham, MA, USA). The qualified DNA was then diluted to 20 ng/mL and stored at 4 °C until further use.

### 2.2. CNV Genotyping

Based on the resequencing data from the goat pan-genome database (http://animal.nwsuaf.edu.cn/code/index.php/panGoat (accessed on 7 March 2022)) and CNVcaller, a read-depth method was used to identify and genotype the CNVs within the goat *Fec^B^* gene. All primers used in this study were described in our previous study [16] (Supplementary Table S1). The CNVs were genotyped using a quantitative PCR (qPCR) technique. The reaction system (10 µL) was amplified and contained 5 µL of 2× SYBR Premix Ex Taq (Takara Biotech, Dalian, China), 0.5 μL of genomic DNA, 0.5 μL of each primer, and 3.5 μL of ddH_2_O [22].

We then used 2 × 2^−ΔCt^ to calculate the relative copy numbers of the CNVs, by which all the CNVs were classified as either loss, normal, or gain, with 2 × 2^−ΔCt^ < 2, 2 × 2^−ΔCt^ = 2, and 2 × 2^−ΔCt^ > 2 representing loss, normal, and gain, respectively.

### 2.3. Association Analysis

To assess whether these five CNVs within the *Fec^B^* gene were associated with growth performance, a linear model was established: Y*_klm__n_*= μ + A*_k_* + G*_l_* + S*_m_* + e*_klm__n_*, where Y*_klmn_* is the observation of morphometric traits, μ is the population mean, A*_k_* is the fixed effect of age, G*_l_* is the fixed effect of genotypes, S*_m_* is the sire effect, and e*_klmn_* is the random error [23]. The normality of the data subjected to statistical analysis was verified. Three types of CNV loci were analyzed using analysis of variance (ANOVA) followed by Dunnett’s multiple comparisons. For CNV loci with two types, Student’s *t*-tests were performed, with *p* < 0.05 indicating statistical significance.

### 2.4. Cluster Analysis, LD Block, and Transcription Factor Binding Prediction

After integrating the goat pan-genome database (version: panGoat_v2 May 20 2019 Assembly, http://animal.nwsuaf.edu.cn/code/index.php/panGoat (accessed on 7 March 2022)) with original CNV data from the east Asian goat population where our selected goat populations were located [24], CNV loci 10 Mb or 1 Mb upstream and downstream from *Fec^B^* (sites with MAF < 0.05 were filtered out) were analyzed for linkage disequilibrium and haplotype block identification. Genotyping clusters of raw CNV data from different populations were identified using Gaussian mixture models (Genotype.py script) in CNVcaller. After typing with CNVcaller [25], the original copy number genotypes of the CNVs were divided into seven types: dd, Ad, AA, AB, BB, BC, and M. Based on the number of copies contained in their genotypes, they were divided into loss (0, copy number < 2), normal (1, copy number = 2), and gain (2, copy number > 2) genotypes. Haploid block detection was performed using the Big-LD algorithm of the gpart package within R software [26]. The visualization was based on the LDblockHeatmap function in gpart, and gene annotation information was obtained from the UCSC genome Browser (panGoat_v2 May 20 2019 Assembly, http://animal.nwsuaf.edu.cn/genomebrowser/cgi-bin/hgTables (accessed on 7 March 2022)).

Additionally, based on the CNV sequence, transcription factors that could bind to the CNVs were predicted using JASPAR (https://jaspar.genereg.net/search?page=3&q=&tax_group=vertebrates&page_size=250&collection=CORE (accessed on 19 January 2022)).

## 3. Results

### 3.1. Detection and Frequency Distribution Analysis

**Figure 1:**
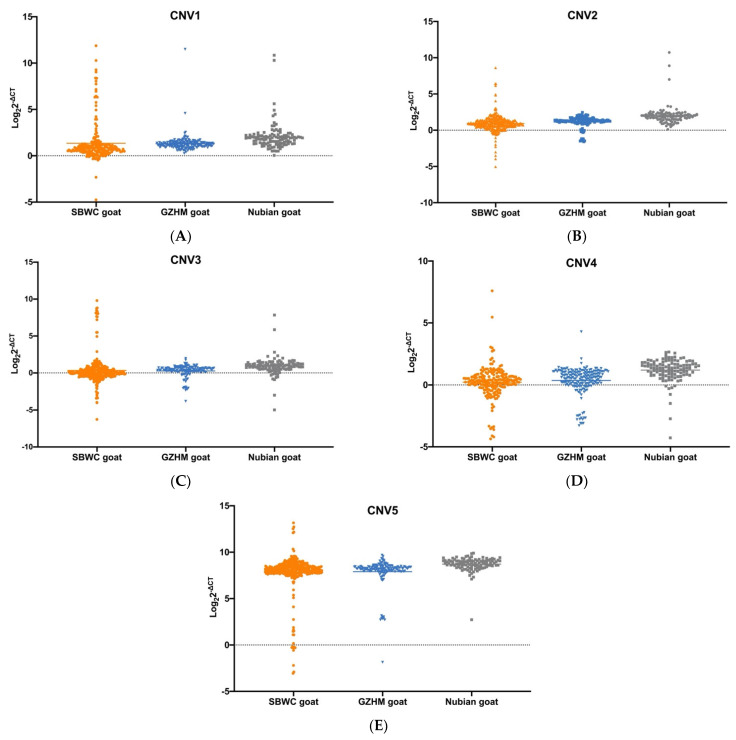
Distribution of different CNVs within the goat *Fec^B^* gene with respect to the vertical axis in SBWC, GZHM, and Nubian goats: (**A**) CNV1, (**B**) CNV2, (**C**) CNV3, (**D**) CNV4, (**E**) CNV5. Note: SBWC goat—Shaanbei white cashmere goats; GZHM goat—Guizhou Heima goat.

Using CNVcaller, five CNVs within the *Fec^B^* gene were identified with heterozygous duplications in several goat breeds (Supplementary Table S2). We further validated the five CNVs in SBWC, GZHM, and Nubian goats using qPCR analysis (Table 1). To estimate the distribution of the five CNVs in the different goat populations, we calculated their frequencies and copy numbers. Our results indicated that CNV1–5 displayed three different types (loss, normal, and gain) in SBWC goats. Notably, gain was the dominant type, with the highest frequency in CNV1–5 within SBWC goats. The same was also observed in GZHM and Nubian goats. Moreover, in GZHM, CNV2, CNV3, and CNV4 had three types, whereas only CNV3 and CNV4 displayed three types in Nubian goats (Table 2). CNV1–5 exhibited different copy number distributions among the goat populations (Figure 1).

### 3.2. Association Analysis

**Figure 2:**
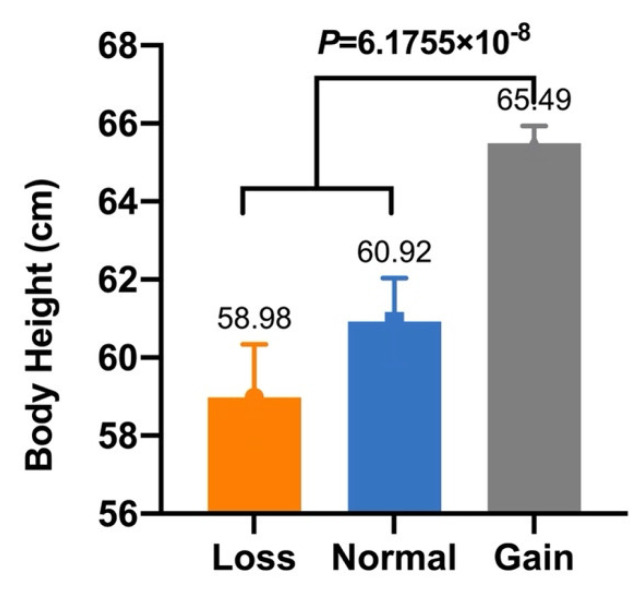
Association analysis between CNV1 and body height in SBWC goats.

**Figure 3:**
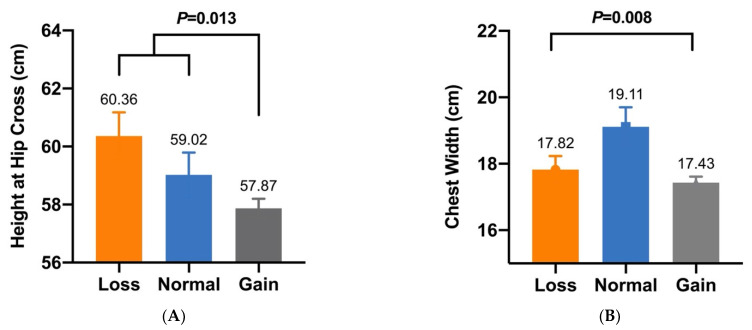
Association analysis between CNV2 and morphometric traits in SBWC goats: (**A**) height at hip cross and (**B**) chest width.

**Figure 4:**
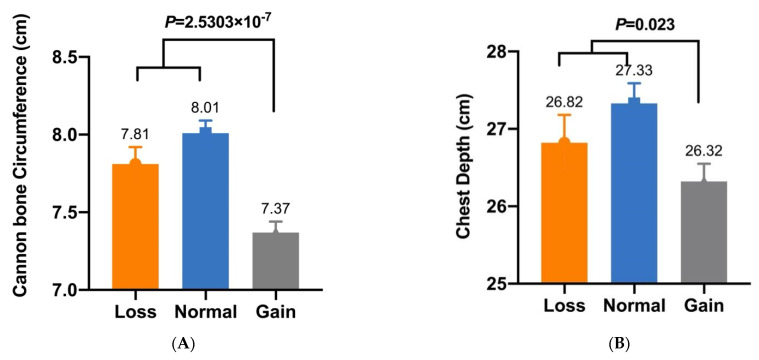
Association analysis between CNV3 and morphometric traits in SBWC goats: (**A**) cannon bone circumference and (**B**) chest depth.

**Figure 5:**
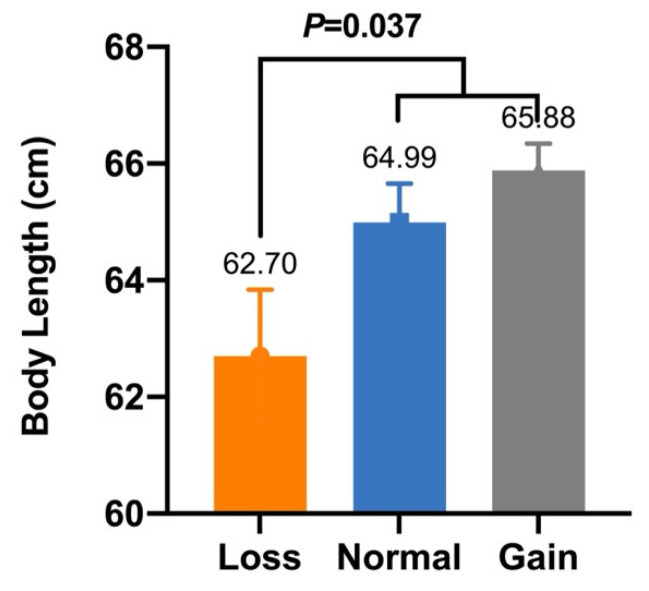
Association analysis between CNV4 and body length in SBWC goats.

**Figure 6:**
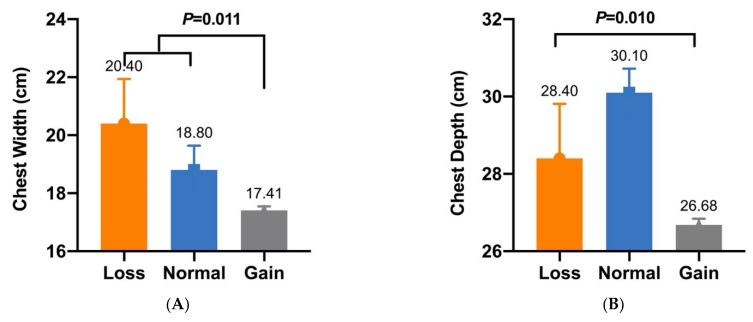
Association analysis between CNV5 and morphometric traits in SBWC goats: (**A**) chest width and (**B**) chest depth.

In our previous study, five CNVs within *Fec^B^* were significantly associated with goat litter sizes [16]. Given that litter size is positively associated with morphometric traits [27], association analyses between these CNVs and morphometric traits were conducted in SBWC, GZHM, and Nubian goats. The association analysis demonstrated that CNV1–5 were significantly associated with morphometric traits in SBWC goats (Table 3). Specifically, CNV1 was significantly associated with body height (*p* = 6.1755 × 10^−8^), and CNV2 had a significant effect on both height at the hip cross (*p* = 0.013) and chest width (*p* = 0.008) (Figure 2 and Figure 3). Furthermore, CNV3 exerted a significant influence on cannon bone circumference (*p* = 2.5303 × 10^−7^) and chest depth (*p* = 0.023), whereas CNV4 was significantly associated with body length (*p* = 0.037) (Figure 4 and Figure 5). Significant associations were also observed between CNV5 and chest width (*p* = 0.011) and depth (*p* = 0.010) (Figure 6); however, no significant association was observed between CNV1–5 and the morphometric traits within GZHM and Nubian goats (Table 4 and Table 5).

### 3.3. Prediction of LD Block and Transcription Factor Binding

**Figure 7:**
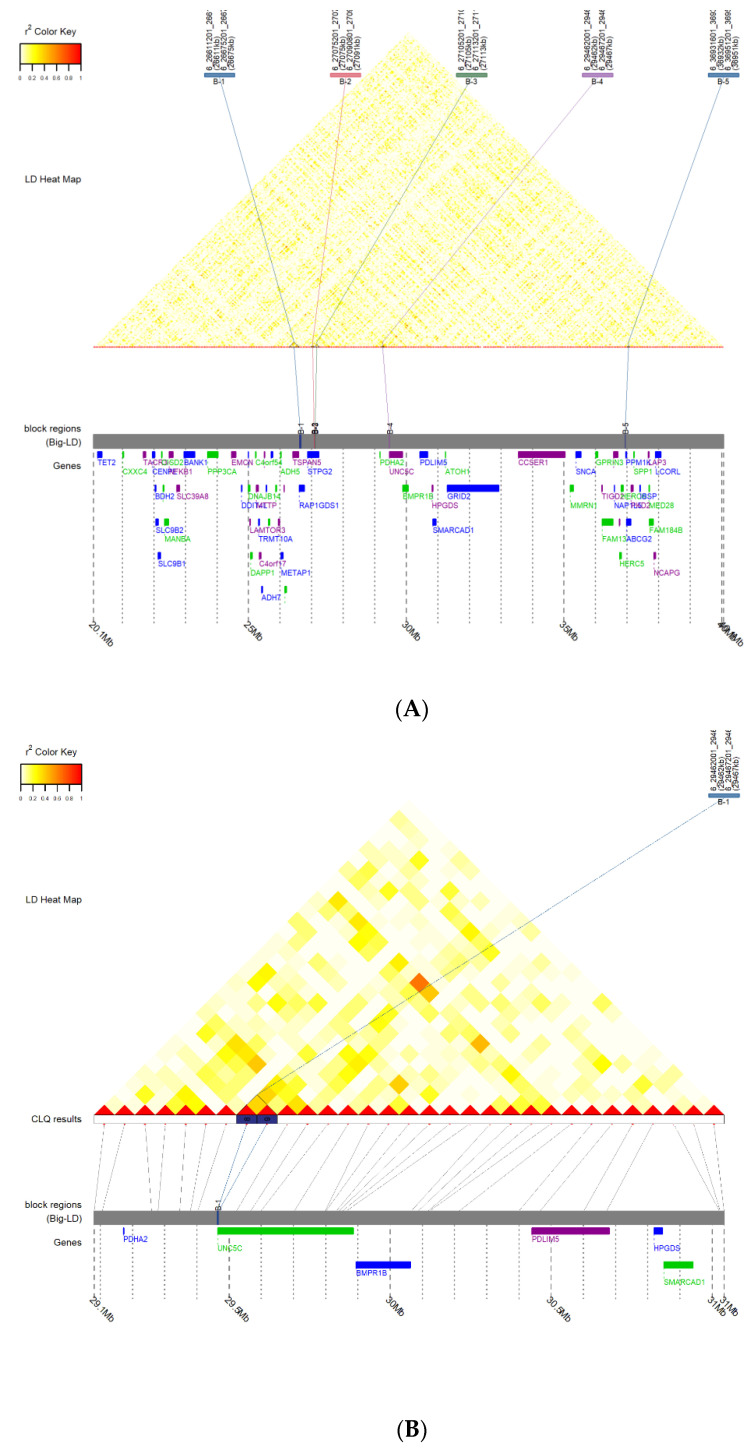
LD block between CNVs at 10 Mb (**A**) or 1 Mb (**B**) distances upstream and downstream of the *Fec^B^* gene.

**Figure 8:**
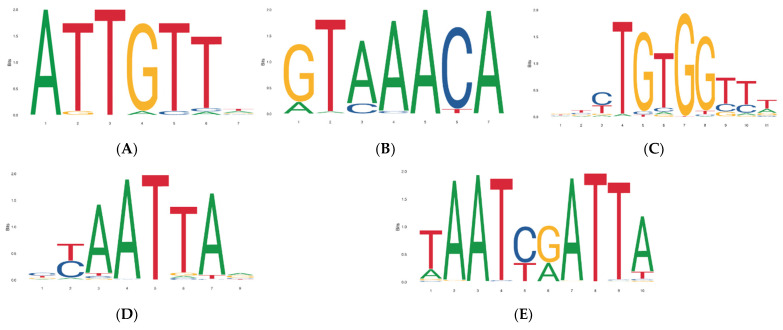
Transcription factor binding prediction of CNVs within the *Fec^B^* gene: (**A**) CNV1, (**B**) CNV2, (**C**) CNV3, (**D**) CNV4, and (**E**) CNV5. Note: this figure represents the distribution of bases on each bp of the motif, and the size of the base is proportional to the corresponding frequency.

To analyze whether the five CNVs were linked to other CNVs and exerted indirect influences, the LD block 10 Mb or 1 Mb upstream and downstream from *Fec^B^* was examined; however, no LD blocks were identified (Figure 7). Since transcription factors (TFs) can bind to a specific DNA sequence, we further predicted transcription factors that might bind to CNV1–5 to exert an indirect influence. Several potential transcription factors were predicted using five CNV sequences and JASPAR, an online prediction tool. Transcription factors with high scores were considered to have a high probability of binding to specific DNA sequences of the five CNVs. Thus, Sox5, Foxo6, Runx1, Shox2, and Pax3, which had the highest scores, could bind to CNV1–5 sequence motifs (Figure 8).

## 4. Discussion

In this study, we used CNVcaller to identify five CNVs within *Fec^B^* in various goat breeds found worldwide. For instance, Nubian goats had obvious heterozygous duplications of the five CNVs, indicating that they contained different genotypes. However, in SBWC goats, only CNV1, CNV3, and CNV4 exhibited different types of duplications. The other two CNVs were normal, which was potentially due to the limited sample size of the resequencing data [25,28]. Notably, the results of this study indicated that these five CNVs had copy number changes other than the normal type in different goat breeds, making subsequent experimental verification meaningful. We then identified these five CNVs in SBWC, GZHM, and Nubian goats. The different distributions of the five CNVs among goat populations may be due to migration [29,30], selection [31,32], mutation [33], random genetic drift [34], and random mating [35]. Moreover, differences in breed and sample size may also have contributed to this finding. In our previous study, we identified five CNVs within the *Fec^B^* gene in SBWC goats and established that CNV3 and CNV5 were significantly associated with litter size [16]. Since there is a positive association between litter size and morphometric traits, we hypothesized that the *Fec^B^* gene also influences growth performance in goats [20].

Furthermore, the association analysis revealed that all five CNVs had a significant influence on morphometric traits in SBWC goats, among which CNV3 and CNV5 were also related to litter size in SBWC goats. Notably, CNV3 demonstrated potential as an effective marker for improving both litter size and growth development. Individuals with the normal type of CNV3 consistently presented superior phenotypes in litter size and morphometric traits. Therefore, selection based on the dominant type should be applied for further breeding work in SBWC goats. For example, if the growth performance and litter size need to be improved, individuals with normal-type CNV3 should be selected. No significant influence of the five CNVs was observed in the GZHM and Nubian goats.

Recently, hypothalamic transcriptome analyses in goats have revealed that *Fec^B^* is differentially expressed between goats with high and low fecundity at the follicular phase, suggesting that the *Fec^B^* gene might be involved in the regulation of hypothalamic function. The hypothalamus regulates the release of growth hormones [36]. Thus, we hypothesized that the *Fec^B^* gene might influence morphometric traits by altering hypothalamic function [37]. Additionally, this gene could inhibit BMP signalling, which is essential for chondrogenesis and for regulating multiple growth plate features, by competitive binding to SPARC-related modular calcium binding 2 (*SMOC2*). Along with this, abnormal BMP pathways induce growth plate defects, resulting in osteochondrodysplasia [38]. Since morphometric traits are controlled by multiple genes and loci, we hypothesized that these five CNVs might be linked to other growth-related polymorphisms and have an indirect effect. However, no LD block was observed within 10 Mb upstream or downstream from *Fec^B^*. Additionally, the sequence of these CNVs might bind to different transcription factors and make it possible to indirectly affect growth and development. Our results indicate that the CNVs could all bind to at least one transcription factor and indirectly perform growth-related functions, such as affecting cartilage development and regeneration [39], skeletal growth [40,41], long-bone development [42], and myogenesis [43,44]. The five CNVs have been predicted to bind to Sox5, Foxo6, Runx1, Shox2, and Pax3, respectively [45,46,47,48]. Furthermore, several genes, such as Runx2 [49], growth differentiation factor 9 (GDF9) [27], and POU class 1 homeobox 1 (POU1F1) [50], have also been reported to simultaneously influence litter size and growth in goats. However, the underlying mechanisms require further investigation. Finally, our findings revealed for the first time that *Fec^B^* was significantly associated with morphometric traits and indicated CNV3 as a potentially effective molecular marker for goat breeding.

## 5. Conclusions

Five CNVs were identified within the *Fec^B^* gene in SBWC, GZHM, and Nubian goats. The five CNVs exhibited different distributions in various goat populations, and gain was the dominant allele with the highest frequency. The five CNVs were significantly associated with morphometric traits in SBWC goats (*p* < 0.05). No significant association was observed between CNV1–5 and the morphometric traits within GZHM and Nubian goats. As expected, we predicted that these five CNVs would have an effect in combination with growth-related TFs. Collectively, these five CNVs exhibited potential for use as DNA markers in future breeding studies.

## Figures and Tables

**Table 1:** Location of CNVs within the goat *Fec^B^* gene on chromosome 6.

Locus	CNV1	CNV2	CNV3	CNV4	CNV5
Start	30,065,201	30,086,001	30,116,801	30,121,201	30,180,401
End	30,067,200	30,088,400	30,118,800	30,123,200	30,182,000
Length (bp)	2000	2400	2000	2000	1600

**Table 2:** Frequency distribution of CNVs within the *Fec^B^* gene in three different goat breeds.

Breed	Locus	Size	Frequency		
			Loss	Normal	Gain
SBWC goat	CNV1	*n* = 215	0.1163 (*n* = 25)	0.1256 (*n* = 27)	0.7581 (*n* = 163)
	CNV2	*n* = 318	0.1384 (*n* = 44)	0.1101 (*n* = 35)	0.7515 (*n* = 239)
	CNV3	*n* = 318	0.1667 (*n* = 53)	0.3019 (*n* = 96)	0.5314 (*n* = 169)
	CNV4	*n* = 213	0.2113 (*n* = 45)	0.3286 (*n* = 70)	0.4601 (*n* = 98)
	CNV5	*n* = 302	0.0232 (*n* = 7)	0.0232 (*n* = 7)	0.9536 (*n* = 288)
GZHM goat	CNV1	*n* = 203	0 (*n* = 0)	0.0049 (*n* = 1)	0.9951 (*n* = 202)
	CNV2	*n* = 158	0.0696 (*n* = 11)	0.0570 (*n* = 9)	0.8734 (*n* = 138)
	CNV3	*n* = 150	0.1133 (*n* = 17)	0.2800 (*n* = 42)	0.6067 (*n* = 91)
	CNV4	*n* = 199	0.1206 (*n* = 24)	0.2663 (*n* = 53)	0.6131 (*n* = 122)
	CNV5	*n* = 158	0.0127 (*n* = 2)	0 (*n* = 0)	0.9873 (*n* = 156)
Nubian goat	CNV1	*n* = 120	0 (*n* = 0)	0.0083 (*n* = 1)	0.9917 (*n* = 119)
	CNV2	*n* = 120	0 (*n* = 0)	0.0083 (*n* = 1)	0.9917 (*n* = 119)
	CNV3	*n* = 116	0.1121 (*n* = 13)	0.0603 (*n* = 7)	0.8276 (*n* = 96)
	CNV4	*n* = 120	0.0417 (*n* = 5)	0.0333 (*n* = 4)	0.9250 (*n* = 111)
	CNV5	*n* = 120	0.0083 (*n* = 1)	0 (*n* = 0)	0.9917 (*n* = 119)

Note: SBWC goat—Shaanbei white cashmere goat; GZHM—Guizhou Heima goat.

**Table 3:** Association analyses between morphometric traits and CNVs in Shaanbei white cashmere goats.

Locus	Trait	Genotype (LSM ± SE)			*p*-Value
		Loss	Normal	Gain	
CNV1	Body Height (cm)	58.98 ± 1.36 ^B^ (*n* = 25)	60.92 ± 1.12 ^B^ (*n* = 27)	65.49 ± 0.45 ^A^ (*n* = 163)	6.1755 × 10^−8^
CNV2	Height at Hip Cross (cm)	60.36 ± 0.78 ^a^ (*n* = 44)	59.02 ± 0.77 ^a^ (*n* = 35)	57.87 ± 0.33 ^b^ (*n* = 239)	0.013
CNV2	Chest Width (cm)	17.82 ± 0.41 ^B^ (*n* = 44)	19.11 ± 0.59 ^A^ (*n* = 35)	17.43 ± 0.18 ^B^ (*n* = 239)	0.008
CNV3	Cannon Bone Circumference (cm)	7.81 ± 0.11 ^A^ (*n* = 53)	8.01 ± 0.08 ^A^ (*n* = 96)	7.37 ± 0.07 ^B^ (*n* = 169)	2.5303 × 10^−7^
CNV3	Chest Depth (cm)	26.82 ± 0.36 ^a^ (*n* = 53)	27.33 ± 0.26 ^a^ (*n* = 96)	26.32 ± 0.23 ^b^ (*n* = 169)	0.023
CNV4	Body Length (cm)	62.70 ± 1.14 ^b^ (*n* = 45)	64.99 ± 0.67 ^a^ (*n* = 70)	65.88 ± 0.46 ^a^ (*n* = 98)	0.037
CNV5	Chest Width (cm)	20.40 ± 1.54 ^a^ (*n* = 7)	18.80 ± 0.84 ^a^ (*n* = 7)	17.41 ± 0.14 ^b^ (*n* = 288)	0.011
CNV5	Chest Depth (cm)	28.40 ± 1.41 ^B^ (*n* = 7)	30.10 ± 0.62 ^A^ (*n* = 7)	26.68 ± 0.16 ^B^ (*n* = 288)	0.010

Note: Values with different letters (A, B/a, b) within the same row differ significantly at *p* < 0.01 or *p* < 0.05.

**Table 4:** Association analyses between morphometric traits and CNVs in Guizhou Heima goats.

Locus	Trait	Genotype (LSM ± SE)			*p*-Value
		Loss	Normal	Gain	
CNV1	Body Weight (kg)	-	47.50 ± 0 (*n* = 1)	32.74 ± 0.89 (*n* = 152)	-
	Body Height (cm)	-	65.00 ± 0 (*n* = 1)	63.46 ± 0.45 (*n* = 181)	-
	Body Length (cm)	-	70.00 ± 0 (*n* = 1)	67.61 ± 0.50 (*n* = 181)	-
	Chest Depth (cm)	-	34.00 ± 0 (*n* = 1)	32.29 ± 0.26 (*n* = 181)	-
	Chest Width (cm)	-	22.00 ± 0 (*n* = 1)	22.27 ± 0.36 (*n* = 181)	-
	Heart Girth (cm)	-	84.00 ± 0 (*n* = 1)	76.20 ± 0.54 (*n* = 181)	-
	Cannon Bone Circumference (cm)	-	8.00 ± 0 (*n* = 1)	7.90 ± 0.03 (*n* = 181)	-
CNV2	Body Weight (kg)	32.5 ± 0.62 (*n* = 10)	40.19 ± 0.14 (*n* = 9)	32.22 ± 0.97 (*n* = 126)	0.120
	Body Height (cm)	66.33 ± 1.40 (*n* = 9)	64.11 ± 0.56 (*n* = 9)	63.91 ± 0.53 (*n* = 123)	0.495
	Body Length (cm)	70.22 ± 1.23 (*n* = 9)	70.22 ± 1.68 (*n* = 9)	68.17 ± 0.66 (*n* = 123)	0.523
	Chest Depth (cm)	34.44 ± 0.50 (*n* = 9)	33.66 ± 0.64 (*n* = 9)	32.44 ± 0.33 (*n* = 123)	0.186
	Chest Width (cm)	22.67 ± 0.50 (*n* = 9)	23.67 ± 0.74 (*n* = 9)	22.02 ± 0.19 (*n* = 123)	0.054
	Heart Girth (cm)	81.00 ± 1.44 (*n* = 9)	80.67 ± 0.69 (*n* = 9)	76.66 ± 0.67 (*n* = 123)	0.082
	Cannon Bone Circumference (cm)	7.88 ± 0.16 (*n* = 9)	8.00 ± 0.16 (*n* = 9)	7.87 ± 0.04 (*n* = 123)	0.564
CNV3	Body Weight (kg)	31.41 ± 0.95 (*n* = 17)	32.55 ± 1.66 (*n* = 35)	32.83 ± 1.28 (*n* = 85)	0.895
	Body Height (cm)	64.42 ± 0.76 (*n* = 14)	64.30 ± 0.81 (*n* = 39)	63.83 ± 0.75 (*n* = 80)	0.926
	Body Length (cm)	69.00 ± 0.85 (*n* = 14)	67.85 ± 1.04 (*n* = 39)	68.46 ± 0.87 (*n* = 80)	0.775
	Chest Depth (cm)	33.78 ± 0.54 (*n* = 14)	32.28 ± 0.54 (*n* = 39)	32.73 ± 0.44 (*n* = 80)	0.166
	Chest Width (cm)	22.71 ± 0.59 (*n* = 14)	22.12 ± 0.33 (*n* = 39)	22.13 ± 0.24 (*n* = 80)	0.642
	Heart Girth (cm)	78.57 ± 1.62 (*n* = 14)	76.07 ± 1.07 (*n* = 39)	77.53 ± 0.91 (*n* = 80)	0.483
	Cannon Bone Circumference (cm)	7.85 ± 0.14 (*n* = 14)	7.87 ± 0.07 (*n* = 39)	7.86 ± 0.05 (*n* = 80)	0.994
CNV4	Body Weight (kg)	33.06 ± 1.95 (*n* = 20)	33.53 ± 1.88 (*n* = 34)	32.71 ± 1.18 (*n* = 96)	0.933
	Body Height (cm)	63.26 ± 1.01 (*n* = 23)	63.86 ± 0.80 (*n* = 49)	63.41 ± 0.62 (*n* = 107)	0.894
	Body Length (cm)	68.00 ± 1.06 (*n* = 23)	67.80 ± 0.98 (*n* = 49)	67.55 ± 0.68 (*n* = 107)	0.950
	Chest Depth (cm)	32.04 ± 0.64 (*n* = 23)	32.59 ± 0.50 (*n* = 49)	32.26 ± 0.35 (*n* = 107)	0.797
	Chest Width (cm)	22.04 ± 0.40 (*n* = 23)	21.88 ± 0.26 (*n* = 49)	22.51 ± 0.60 (*n* = 107)	0.738
	Heart Girth (cm)	76.26 ± 1.33 (*n* = 23)	76.20 ± 1.12 (*n* = 49)	76.27 ± 0.72 (*n* = 107)	0.999
	Cannon Bone Circumference (cm)	7.87 ± 0.09 (*n* = 23)	7.84 ± 0.08 (*n* = 49)	7.88 ± 0.04 (*n* = 107)	0.764
CNV5	Body Weight (kg)	29.30 ± 5.30 (*n* = 2)	-	32.95 ± 0.93 (*n* = 143)	-
	Body Height (cm)	54.00 ± 0 (*n* = 1)	-	64.19 ± 0.49 (*n* = 140)	-
	Body Length (cm)	60.00 ± 0 (*n* = 1)	-	68.49 ± 0.59 (*n* = 140)	-
	Chest Depth (cm)	27.00 ± 0 (*n* = 1)	-	32.78 ± 0.30 (*n* = 140)	-
	Chest Width (cm)	20.00 ± 0 (*n* = 1)	-	22.23 ± 0.18 (*n* = 140)	-
	Heart Girth (cm)	66.00 ± 0 (*n* = 1)	-	77.32 ± 0.62 (*n* = 140)	-
	Cannon Bone Circumference (cm)	8.00 ± 0 (*n* = 1)	-	7.87 ± 0.04 (*n* = 140)	-

Note: Loci containing only two types, as well as one CNV type with a sample size of less than 5, were not included in the statistical analysis.

**Table 5:** Association analyses between morphometric traits and CNVs in Nubian goats.

Locus	Trait	Genotype (LSM ± SE)			*p*-Value
		Loss	Normal	Gain	
CNV1	Body Weight (kg)	-	47.55 ± 0.55 (*n* = 2)	50.29 ± 0.98 (*n* = 118)	-
	Body Height (cm)	-	75.15 ± 0.35 (*n* = 2)	72.23 ± 0.50 (*n* = 118)	-
	Body Length (cm)	-	70.90 ± 1.30 (*n* = 2)	65.05 ± 0.51 (*n* = 118)	-
	Heart Girth (cm)	-	89.50 ± 1.90 (*n* = 2)	87.34 ± 0.78 (*n* = 118)	-
	Chest Width (cm)	-	21.15 ± 0.25 (*n* = 2)	21.45 ± 0.39 (*n* = 118)	-
	Chest Depth (cm)	-	32.50 ± 2.00 (*n* = 2)	34.91 ± 0.44 (*n* = 118)	-
	Cannon Bone Circumference (cm)	-	10.50 ± 0.80 (*n* = 2)	10.41 ± 0.71 (*n* = 118)	-
CNV2	Body Weight (kg)	64.50 ± 0 (*n* = 1)	44.52 ± 3.55 (*n* = 4)	50.38 ± 1.00 (*n* = 112)	-
	Body Height (cm)	73.00 ± 0 (*n* = 1)	70.75 ± 1.02 (*n* = 4)	72.18 ± 0.50 (*n* = 112)	-
	Body Length (cm)	68.50 ± 0 (*n* = 1)	68.30 ± 1.86 (*n* = 4)	64.95 ± 0.53 (*n* = 112)	-
	Heart Girth (cm)	97.30 ± 0 (*n* = 1)	88.58 ± 1.16 (*n* = 4)	87.11 ± 0.80 (*n* = 112)	-
	Chest Width (cm)	29.10 ± 0 (*n* = 1)	21.60 ± 1.35 (*n* = 4)	21.30 ± 0.41 (*n* = 112)	-
	Chest Depth (cm)	41.00 ± 0 (*n* = 1)	32.80 ± 3.80 (*n* = 4)	34.74 ± 0.43 (*n* = 112)	-
	Cannon Bone Circumference (cm)	10.50 ± 0 (*n* = 1)	9.88 ± 0.55 (*n* = 4)	10.42 ± 0.75 (*n* = 112)	-
CNV3	Body Weight (kg)	46.21 ± 1.46 (*n* = 13)	52.53 ± 0.43 (*n* = 7)	50.70 ± 1.02 (*n* = 96)	0.309
	Body Height (cm)	69.77 ± 1.90 (*n* = 13)	69.75 ± 2.55 (*n* = 7)	72.56 ± 0.50 (*n* = 96)	0.106
	Body Length (cm)	63.95 ± 1.96 (*n* = 13)	61.94 ± 0.78 (*n* = 7)	65.45 ± 0.54 (*n* = 96)	0.216
	Heart Girth (cm)	32.33 ± 1.14 (*n* = 13)	32.56 ± 1.58 (*n* = 7)	35.13 ± 0.41 (*n* = 96)	0.417
	Chest Width (cm)	21.12 ± 1.24 (*n* = 13)	18.75 ± 1.16 (*n* = 7)	21.56 ± 0.44 (*n* = 96)	0.251
	Chest Depth (cm)	85.43 ± 1.50 (*n* = 13)	83.96 ± 1.87 (*n* = 7)	97.58 ± 0.84 (*n* = 96)	0.060
	Cannon Bone Circumference (cm)	9.51 ± 0.25 (*n* = 13)	9.41 ± 0.20 (*n* = 7)	10.60 ± 0.87 (*n* = 96)	0.845
CNV4	Body Weight (kg)	50.43 ± 3.16 (*n* = 6)	47.77 ± 3.71 (*n* = 3)	50.30 ± 1.01 (*n* = 111)	-
	Body Height (cm)	69.77 ± 2.44 (*n* = 6)	69.80 ± 1.61 (*n* = 3)	72.48 ± 0.49 (*n* = 111)	-
	Body Length (cm)	65.88 ± 2.80 (*n* = 6)	67.80 ± 2.66 (*n* = 3)	65.04 ± 0.53 (*n* = 111)	-
	Heart Girth (cm)	88.18 ± 4.07 (*n* = 6)	84.33 ± 5.58 (*n* = 3)	87.38 ± 0.77 (*n* = 111)	-
	Chest Width (cm)	21.91 ± 2.01 (*n* = 6)	18.37 ± 3.91 (*n* = 3)	21.50 ± 0.40 (*n* = 111)	-
	Chest Depth (cm)	34.53 ± 2.78 (*n* = 6)	32.07 ± 1.57 (*n* = 3)	34.96 ± 0.44 (*n* = 111)	-
	Cannon Bone Circumference	9.67 ± 0.25 (*n* = 6)	10.30 ± 1.15 (*n* = 3)	10.46 ± 0.76 (*n* = 111)	-
CNV5	Body Weight (kg)	67.10 ± 0 (*n* = 1)	-	50.01 ± 1.00 (*n* = 108)	-
	Body Height (cm)	71.20 ± 0 (*n* = 1)	-	72.05 ± 0.50 (*n* = 108)	-
	Body Length (cm)	67.50 ± 0 (*n* = 1)	-	65.04 ± 0.53 (*n* = 108)	-
	Heart Girth (cm)	90.80 ± 0 (*n* = 1)	-	87.04 ± 0.80 (*n* = 108)	-
	Chest Width (cm)	20.70 ± 0 (*n* = 1)	-	21.27 ± 0.41 (*n* = 108)	-
	Chest Depth (cm)	34.30 ± 0 (*n* = 1)	-	34.61 ± 0.42 (*n* = 108)	-
	Cannon Bone Circumference (cm)	9.00 ± 0 (*n* = 1)	-	10.46 ± 0.78 (*n* = 108)	-

Note: Loci containing only two types, as well as one CNV type with a sample size of less than 5, were not included in the statistical analysis.

## Data Availability

The data presented in this study are available in the article.

## Supplementary Materials

The following supporting information can be downloaded at: https://www.mdpi.com/article/10.3390/ani12121547/s1, Table S1: The primers used in this study; Table S2: Result of CNVcaller.
