# Supplementary material for: Are Copy Number Variations within the FecB Gene Significantly Associated with Morphometric Traits in Goats?
Source: Animals (Basel). 2022 Jun 15;12(12):1547. doi: 10.3390/ani12121547 (PMC9219420; doi:10.3390/ani12121547)
Supplement: Supplementary file 1 [file animals-12-01547-s001.zip › animals-1724592-supplementary Table S1.pdf]

Supplementary Table S1. The primers used in this study.

Primers	Primer sequence (5′ to 3′)	Tm (°C)	Length	Application
CNV1-F	TGAAAACAAGGAGGCAAGGAA	57.97	141bp	CNV detection
CNV1-R	TAACCCTTCATCACCTTTCTCC	57.43		
CNV2-F	AGAGGCTGAGGTCTAAATTGTT	57.08	158bp	CNV detection
CNV2-R	GACTGCTCATTTGTTGGTGGG	59.73		
CNV3-F	CAGATTTCAGCCTTTGCGGG	59.83	111bp	CNV detection
CNV3-R	TTGGGGCAGTCAGGAAAGAG	59.60		
CNV4-F	CAGTCGTATCCTGGCACTGA	59.18	165bp	CNV detection
CNV4-R	TGCCTTTAGGTCAGTGGGAAC	59.93		
CNV5-F	CCAAGGTAACCCAGAACTAGACACA	58.60	200bp	CNV detection
CNV5-R	ACGACGACATCAGAGGGAGACA	58.90		
MC1R-F	GGCCTGAGAGGGGAATCACA	61.27	126bp	Internal Control
MC1R-R	AGTGGGTCTCTGGATGGAGG	60.33		

Note: All primers were referenced in our previous study [1].

Reference

1. Bi, Y.; Feng, W.; Kang, Y.; Wang, K.; Yang, Y.; Qu, L.; Chen, H.; Lan, X.; Pan, C. Detection of mRNA expression and copy number variations within the goat *Fec*^{B} gene associated with litter size. Frontiers in Veterinary Science 2021, 8, 758705. doi:10.3389/fvets.2021.758705.
